# Discrepancies in breast cancer’s oncological outcomes between public and private institutions in the southeast region of Brazil: a retrospective cohort study

**DOI:** 10.3389/fonc.2023.1169982

**Published:** 2023-06-27

**Authors:** Diocésio Alves Pinto Andrade, Ana Carolina Veneziani, Carlos Eduardo Paiva, Ricardo dos Reis, Carlos Alberto Fruet Filho, André Octávio Nicolau Sanches, Alison Wagner Azevedo Barroso, Alessandra Caroline Moretto Carbinatto Paz, Georgia Cristina de Mello Kons, Daniel D’Almeida Preto, Maria Carolina Bogoni Budib, Maria Augusta Safro, Gustavo Sanches Faria Pinto, João Paolo Bilibio, Cristiano de Pádua Souza

**Affiliations:** ^1^ Clinical Oncology Department, InORP Oncoclínicas Group, Oncology Institute of Ribeirão Preto, Ribeirão Preto, Brazil; ^2^ Medical Oncology Department, Princess Margareth Cancer Center, Toronto, ON, Canada; ^3^ Clinical Oncology Department, Division of Breast and Gynecologic, Barretos Cancer Hospital, Barretos, Brazil; ^4^ Gynecologic Oncology Department, Barretos Cancer Hospital, Barretos, Brazil; ^5^ Medical Oncology Department, UNIMED, Uberaba, Brazil; ^6^ Medical Oncology Department, Liga Contra Cancer, Natal, Brazil; ^7^ Medical Oncology Department, Amaral Carvalho Hospital, Jaú, Brazil; ^8^ Medical Oncology Department, Santa Casa de Misericórdia, Curitiba, Brazil; ^9^ Clinical Oncology Department, Division of Urology, Barretos Cancer Hospital, Barretos, Brazil; ^10^ Medical Oncology Department, Campo Grande Cancer Institute, Campo Grande, Brazil; ^11^ Medical Oncology Department, BC Cancer Agency, Vancouver, BC, Canada; ^12^ Medical Oncology Department, Hospital de Base, São José do Rio Preto, Brazil; ^13^ UNIFEBE, Brusque, Brazil

**Keywords:** breast neoplasms, Brazil, public treatment setting, private treatment setting, oncological outcomes

## Abstract

**Background:**

Brazil is a middle-income country with inequalities in its healthcare system. The disparities between public and private services affect the diagnosis and treatment of patients with breast cancer. The aim of this study is to assess whether disease-free survival (DFS) and overall survival (OS) are different in public and private specialized centers.

**Patient and methods:**

A retrospective cohort study with 1,545 breast cancer patients diagnosed from 2003 to 2011 at Barretos Cancer Hospital—BCH (public group, N = 1,408) and InORP Oncoclinicas (private group, N = 137) was conducted. A 1:1 propensity score matching (PSM) analysis was used to adjust the differences between the groups’ characteristics (n = 137 in each group).

**Results:**

The median age at diagnosis was 54.4 years. Estimated DFS rates at 1, 5, and 10 years were 96.0%, 71.8%, and 59.6%, respectively, at BCH and 97.8%, 86.9%, and 78%, respectively, at InORP (HR: 2.09; 95% confidence interval [CI], 1.41–3.10; *p* < 0.0001). Estimated OS rates at 1, 5, and 10 years were 98.1%, 78.5%, and 65.4%, respectively, at BCH and 99.3%, 94.5%, and 91.9%, respectively, at InORP (HR: 3.84; 95% CI, 2.16–6.82; *p* < 0.0001). After adjustment by PSM, DFS and OS results in 1, 3, and 5 years remained worse in the public service compared to the private service.

**Conclusion:**

Patients treated in a public center have worse DFS and OS after a follow-up period of more than 5 years. These results were corroborated after carrying out the PSM.

## Introduction

Breast cancer is the most common female type of cancer worldwide and is the leading cause of cancer-related mortality among this population. In Brazil, 73,610 new cases are estimated for 2023 (1). Brazil is the largest country in South America, composed of 27 administrative divisions distributed into five regions (north, northeast, center-west, southeast, and south). Brazil’s healthcare system is divided into two sectors: public (named Sistema Único de Saúde [SUS]) and private (2). Approximately 80% of Brazil’s total population (currently at over 212 million people) are users of the public health system (3). Nearly 47 million (22.4%) have access to the private system through insurance or health plans. The highest concentration of health-insured people is in the southeast region of the country (4). Therefore, it is evident that there are important social and economic imbalances between regions. For example, the proportion of patients who never had a screening mammogram is higher in the northern and northeast regions, which are resource-limited compared to those in the south and southeast regions. Furthermore, the highest rates of breast cancer are concentrated in the south and southeast regions (1). These numbers may reflect the heterogeneous healthcare resources between regions.

The AMAZONA III, a prospective observational study including data from all Brazilian regions, showed that breast cancer patients from the public health system were diagnosed at more advanced stages and aggressive subtypes than privately insured patients (5). One of the hypotheses is that breast cancer screening is carried out differently between the public and private sectors. In the public services, biannual mammography for women aged 50–69 years is recommended; in the private sector, this exam is performed annually starting at 40 years (6). Similarly, Gonzaga and colleagues demonstrated differences in BC-related mortality rates according to the geographic region of Brazil. Overall, states of the federation with a higher Human Development Index (HDI), such as the southeast region, have lower rates of breast cancer mortality (7).

The state with the highest HDI in Brazil is São Paulo (located in the southeast), which has the most developed healthcare system. In addition to 20% of the national population residing there, São Paulo also attracts thousands of patients from all over the country who require cancer care (8). Studies have previously investigated the influence of public and private practices on breast cancer outcomes considering the entire country (5, 9). However, Brazil is highly heterogeneous socially and economically. No information is known regarding sociodemographics, treatment patterns, and outcomes of breast cancer patients between public and private institutions in the countryside of São Paulo state.

Therefore, our retrospective study aimed to characterize the impact of insurance status (public and private institutions) on clinical oncological outcomes of breast cancer patients in the southeast of Brazil, more specifically in the countryside of São Paulo state, which has a more structured and homogeneous healthcare system.

## Patient and methods

### Structure of the participating reference cancer centers

The cohort of patients was established from two institutions located in the countryside of São Paulo state. The first one was Barretos Cancer Hospital (BCH), which is one the largest tertiary cancer care center in Brazil. The other institution was the InORP Oncoclinicas Group (InORP), which is a private oncology clinic that treats patients with different types of private healthcare benefit plans. A database chart review was built in the RedCap platform from both institutions. The patient information was manually collected by the study investigators from the medical charts, which included institution, age at diagnosis, gender, clinical and pathological features, treatment modalities (surgery, radiotherapy, chemotherapy, and endocrine therapy), disease recurrence or progression, and survival data. Adult women (aged >18 years) with pathologically confirmed stages ≤ III breast cancer between January 2003 to December 2011 were included. Patients registered before 2009 were staged by American Joint Committee on Cancer (AJCC) TNM 6th edition (10). After 2009, patients were staged by AJCC TNM 7th edition (11). All histologies were accepted. All patients from Barretos Cancer Hospital were considered public health coverage, and those from InORP Oncoclinicas Group were private health coverage.

Breast cancer subtypes were defined using estrogen receptor (ER), progesterone receptor (PR), and human epidermal growth factor receptor 2 (HER2) status in immunohistochemistry from local pathology laboratories (12). Although a central pathology review was not performed, all associated laboratories perform quality controls concerning their immunohistochemistry analysis. Three breast cancer subtypes were defined: luminal subtype—ER positive and/or PR positive, HER2 negative; HER2 subtype—ER/PR positive or negative, HER2 positive; triple-negative subtype (TN)—ER negative, PR negative, and HER2 negative. We did not consider differences between Luminal A and B because we could not retrieve Ki67 results in most of the patients.

### Outcomes

The primary outcomes were disease-free survival (DFS) and overall survival (OS). DFS was defined as the time from the diagnosis until the first recurrence, death, or last contact. OS was defined as the time from the diagnosis until death related to any cause or last contact. Patients who were lost to follow-up were censored at the last contact.

### Statistical analysis

Data analyses were performed using relative and absolute frequencies. Subsequently, associations were performed using the chi-square test or Fisher’s exact test. The Kaplan–Meier method was used to estimate DFS and OS, and the differences were compared using log-rank tests in univariate analysis. Effects of where patients had their treatments (Barretos Cancer Hospital *vs.* InORP Oncoclinicas Group) on DFS and OS were calculated using Cox regression models with adjustment for age, body mass index (BMI), stage, breast cancer subtype, and histologic grade. All variables with *p*-values ≤0.2 in the univariate analysis were entered into multivariate analysis conducted using Cox proportional hazards modeling. All tests were two-sided, and a *p-*value <0.05 was considered statistically significant.

Owing to the retrospective nature of the study design and the possible allocation biases from the retrospective comparison between Barretos Cancer Hospital and InORP Oncoclinicas Group cohorts, we performed a propensity score matching (PSM) analysis (13). The PSM was used to minimize potential selection bias. A propensity score was developed through a multivariable logistic regression model adjusting for stage disease at diagnosis. A 1:1 control group with patients from Barretos Cancer Hospital was applied. Each patient in stages I, II, and III from the InORP Oncoclinicas Group was matched with one patient treated from Barretos Cancer Hospital who had the closest estimated propensity score. Survival analysis, DFS, and OS were performed for both the whole study and the PSM population.

Due to the difference in the sample between the groups (approximately 1 case from InORP Oncoclinicas Group and 10 cases from Barretos Cancer Hospital), we performed a power analysis for each of the significant results of our study: histology type, Fisher’s test 85.48% and Mid-p test 87.86%; clinical stage, Fisher’s test 99.94% and Mid-p test 99.96%; type of surgery, Fisher’s test 98.45% and Mid-p test 98.79%; type of chemotherapy, Fisher’s test 53.51% and Mid-p test 58.09%.

Study data were collected and managed using REDCap (Research Electronic Data Capture provided by Barretos Cancer Hospital) (14). Data analysis was performed using IBM Statistical Package for the Social Sciences (SPSS) database version 27.0 (SPSS, Chicago, IL, USA).

This study was conducted following the ethical principles of the Declaration of Helsinki, and the Barretos Cancer Hospital Ethical Review Board approved it in February 2017 (reference number 1.928.867).

## Results

A total of 1,545 patients were included in the study. Their baseline characteristics are listed in **Table 1**. The majority of patients were publicly insured, with 1,408 (91.1%) from Barretos Cancer Hospital and 137 (8.9%) from InORP Oncoclinicas Group. Median age and BMI at diagnosis were, respectively, 54.4 years (range 20.8–95.8) and 27.1 (range 13.3–53.6), which were similar between both institutions.

**Table 1:** Clinical and pathological features for all patients and according to place of treatment.

	All patients N = 1,545	BCH N = 1,408	InORP N = 137	*p*-Value
Age (p25–p75)	45.7–54.3	45.7–63.0	44.7–62.4	0.588
BMI (p25–p75)	23.8–30.7	23.9–30.7	23.3–30.7	0.828
Histology type				
Ductal (%)	1,336 (87.0%)	1,231 (88.0%)	105 (76.6%)	**<0.001**
Lobular (%)	118 (7.7%)	95 (6.8%)	23 (16.8%)	
Others (%)	82 (5.3%)	73 (5.2%)	9 (6.6%)	
Clinical stage at diagnosis				
I (%)	379 (24.5%)	306 (21.7%)	73 (53.3%)	**<0.001**
II (%)	574 (37.2%)	534 (37.9%)	40 (29.2%)	
III (%)	592 (38.3%)	568 (40.3%)	24 (17.5%)	
Histologic grade				
G1 (%)	169 (10.9%)	150 (10.7%)	19 (13.9%)	0.145
G2 (%)	846 (54.8%)	772 (54.8%)	74 (54.0%)	
G3 (%)	482 (31.2%)	450 (32.0%)	32 (23.4%)	
Gx (%)	48 (3.1%)	36 (2.5%)	12 (8.7%)	
Subtype				
Luminal (%)	898 (62.6%)	805 (61.9%)	93 (69.4%)	0.147
HER2 (%)	311 (21.7%)	284 (21.8%)	27 (20.1%)	
Triple negative (%)	225 (15.7%)	211 (16.2%)	14 (10.4%)	

BCH, Barretos Cancer Hospital; InORP, InORP Oncoclinicas Group; BMI, body mass index; HER2, human epidermal growth factor receptor 2.Bold values are statistically significant.

Regarding the clinicopathological characteristics at diagnosis, most patients had more invasive ductal carcinoma histology (87.0%), histologic grade 2 (56.5%), and TNM stages II and III (37.2% and 38.3%, respectively) in the global population. The luminal subtype was the most common breast cancer subtype (62.6%), followed by the HER2 subtype (21.7%) and TN subtype (15.7%); the distribution was similar between the two sites.

The institution where the patients underwent their treatment was associated with breast cancer stage at diagnosis. Stage I patients were more prevalent at InORP Oncoclinicas Group (53.3% *versus* 21.7%; *p* < 0.001), whereas stage III patients were more common at Barretos Cancer Hospital (40.3% *versus* 17.5%; *p* < 0.001).

The treatment modalities are shown in **Table 2**. The proportional number of patients who had undergone conservative breast surgery was significantly higher at private services compared with public services (71.5% *versus* 49.8%; *p* < 0.001). Adjuvant radiotherapy was given equally in both institutions (87.7% *versus* 84.4%; *p* = 0.283). Considering the two centers’ patients, 84.1% of stage I, 87.6% of stage II, and 89.2% of stage III breast cancer received adjuvant radiotherapy.

**Table 2:** Locoregional and systemic treatments received by all patients according to the place of treatment.

	All patients N = 1,545	BCH N = 1,408	InORP N = 137	*p*-Value
Surgery (%)	1,500 (97.1%)	1,363 (96.8%)	137 (100%)	0.033
Mastectomy	701 (45.4%)	662 (47.0%)	39 (28.5%)	**<0.001**
Conservative	799 (51.7%)	701 (49.8%)	98 (71.5%)	
Unknown	45 (2.9%)	45 (3.2%)	0	
Radiotherapy (%)	1,315 (85.1%)	1,201 (85.3%)	114 (83.2%)	0.283
Chemotherapy (%)	1,182 (76.5%)	1,073 (76.2%)	109 (79.6%)	0.491
Neoadjuvant (%)	358 (30.3%)	336 (31.3%)	22 (20.2%)	**0.016**
Adjuvant (%)	824 (69.7%)	737 (68.6%)	87 (79.8%)	
CMF (%)	158 (13.4%)	153 (14.3%)	5 (4.6%)	**<0.001**
Anthracyclines (%)	380 (32.1%)	359 (33.5%)	21 (19.3%)	
Taxanes (%)	46 (3.9%)	26 (2.4%)	20 (18.3%)	
Anthracyclines/taxanes (%)	560 (47.4%)	508 (47.3%)	52 (47.7%)	
Others (%)	38 (3.2%)	27 (2.5%)	11 (10.1%)	
Trastuzumab (%) (HER2+)	141 (45.3%)	124 (43.7%)	17 (62.9%)	0.054

BCH, Barretos Cancer Hospital; InORP, InORP Oncoclinicas Group; CMF, cyclophosphamide plus methotrexate plus fluorouracil; HER2, human epidermal growth factor receptor 2.Bold values are statistically significant.

Both sites performed almost the same rate of chemotherapy (76.2% in Barretos Cancer Hospital *versus* 79.6% in InORP Oncoclinicas Group; *p* = 0.491). Most patients received adjuvant chemotherapy (68.6% in public *versus* 79.8% in private services), and neoadjuvant treatment was performed in 31.3% of Barretos Cancer Hospital patients *versus* 20.2% at InORP Oncoclinicas Group (*p* = 0.016). Overall, anthracyclines plus taxanes were the most used chemotherapy combination (47.4%), followed by anthracyclines single schedule (32.1%) and CMF (cyclophosphamide plus methotrexate plus fluorouracil; 13.4%) (**Table 2**). Although historically the regimen of chemotherapy has been associated with increased DFS and OS, we could not calculate the number of events to show any difference in this cohort. Targeted treatment with trastuzumab for HER2-positive breast cancer subtype was given to 43.7% and 62.9% (*p* = 0.054) of Barretos Cancer Hospital and InORP Oncoclinicas Group patients, respectively (**Table 2**).

Regarding endocrine therapy, 93.8% of patients with luminal subtype breast cancer from both institutions received this treatment modality. Tamoxifen was more often used for both sites in all stages (521 patients), followed by aromatase inhibitors alone (269 patients) or in sequence after tamoxifen (52 patients). Proportionally, patients from private and public services received the same rate of adjuvant aromatase inhibitors [29 (31.9%) *vs.* 240 (32.0%); *p* = 0.982] within all early stages.

**Figure 1:**
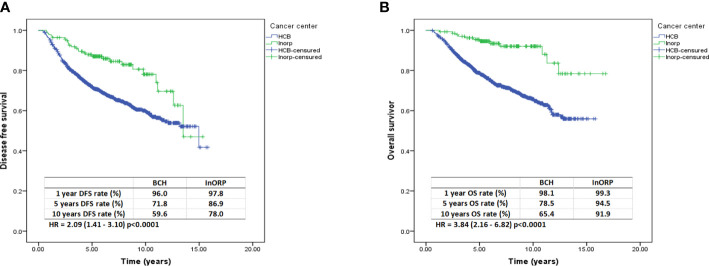
Kaplan–Meier curves for DFS **(A)** and OS **(B)**. DFS, disease-free survival; OS, overall survival.

After a median follow-up of 5.8 years from diagnosis, 357 (23.1%) patients had relapsed (334 [23.7%] from BCH and 23 [16.8%] from InORP), and 402 (26.0%) had died (390 [27.7%] from BCH and 12 [8.8%] from InORP). The Kaplan–Meier estimates of DFS at 1, 5, and 10 years were 96.0%, 71.8%, and 59.6%, respectively, at Barretos Cancer Hospital and 97.8%, 86.9%, and 78%, respectively, at InORP Oncoclinicas Group (HR: 2.09; 95% confidence interval [CI], 1.41–3.10; *p* < 0.0001; **Figure 1**). The Kaplan–Meier estimates of OS at 1, 5, and 10 years were 98.1%, 78.5%, and 65.4%, respectively, at Barretos Cancer Hospital and 99.3%, 94.5%, and 91.9%, respectively, at InORP Oncoclinicas Group (HR: 3.84; 95% CI, 2.16–6.82; *p* < 0.0001; **Figure 1**).

The analysis by staging I, II, and III, for both DFS and OS, showed a statistically significant difference in favor of the private service, as shown in the Supplementary Material (**Figures S1**, **S2**).

Adjusted multivariate analysis (**Table 3**) showed that patients treated at the public health system had worse OS [HR 2.38; 95% CI, 1.30–4.38; *p* = 0.05]. This outcome was associated with more aggressive patterns such as stage II [HR 1.64; 95% CI, 1.09–2.45; *p* = 0.017] or stage III disease [HR 4.90; 95% CI, 3.36–7.15; *p* < 0.001], TN subtype [HR 1.83; 95% CI, 1.41–2.37; *p* < 0.001], and grade 2 [HR 1.99; 95% CI, 1.18–3.39; *p* = 0.010] or grade 3 [HR 2.21; 95% CI, 1.28–3.82; *p* = 0.004].

**Table 3:** Multivariate analysis for DFS and OS.

	DFS		OS	
	HR (95% CI)	*p*-Value	HR (95% CI)	*p*-Value
InORP (ref.)				
BCH	1.13 (0.72–1.80)	0.594	2.38 (1.30–4.38)	**0.005**
Age	0.99 (0.99–1.00)	0.221	1.026 (1.01–1.04)	**<0.001**
Stage				
I (ref.)				
II	1.36 (0.89–2.07)	0.158	1.64 (1.09–2.45)	**0.017**
III	4.67 (3.17–6.90)	<0.001	4.90 (3.36–7.15)	**<0.001**
Subtype				
Luminal (ref.)				
HER2	1.06 (0.81–1.39)	0.679	1.17 (0.90–1.52)	0.239
TN	1.48 (1.11–1.96)	0.006	1.83 (1.41–2.37)	**<0.001**
Grade				
G1 (ref.)				
G2	3.31 (1.62–6.75)	0.001	1.99 (1.18–3.39)	**0.010**
G3	4.45 (2.16–9.18)	<0.001	2.21 (1.28–3.82)	**0.004**

BCH, Barretos Cancer Hospital; InORP, InORP Oncoclinicas Group; DFS, disease-free survival; OS, overall survival; TN, triple negative; HR, hazard ratio; CI, confidence interval; ref., reference; HER2, human epidermal growth factor receptor 2.Bold values are statistically significant.

**Figure 2:**
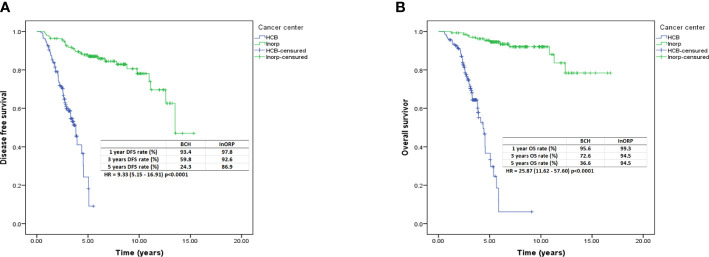
Kaplan–Meier curves for DFS **(A)** and OS **(B)** after PSM analysis. DFS, disease-free survival; OS, overall survival; PSM, propensity score matching.

Additionally, 274 patients were analyzed after PSM (137 patients from Barretos Cancer Hospital were matched with 137 patients from InORP Oncoclinicas Group). No differences emerged between the clinical–pathological stage of the two groups. The Kaplan–Meier estimates of DFS at 1, 3, and 5 years were 93.4%, 59.8%, and 24.3%, respectively, at BCH and 97.8%, 92.6%, and 86.9%, respectively, at InORP (HR: 9.33; 95% CI, 5.15–16.91; *p* < 0.0001; **Figure 2**). The Kaplan–Meier estimates of OS at 1, 3, and 5 years were 95.6%, 72.6%, and 36.6%, respectively, at BCH and 99.3%, 94.5%, and 94.5%, respectively, at InORP (HR: 25.87; 95% CI, 11.62–57.60; *p* < 0.0001; **Figure 2**).

## Discussion

This study assessed the clinical characteristics and outcomes in patients with breast cancer treated in public and private oncologic centers in São Paulo, the most developed state in Brazil. We demonstrated that DFS and OS, after more than 5 years of follow-up, were worse in a public center. Furthermore, after the adjustment of the populations by PSM, both outcomes remained worse in the public scenario.

Performing this analysis in patients from the state of São Paulo, but from different services (public and private), we excluded the territorial bias that may exist in the country. Moreover, it is important to highlight that the two centers are only 75 miles apart. Considering Barretos Cancer Hospital (also known as “Hospital de Amor”) as a reference public cancer center in Latin America, in terms of both clinical assistance and research, the discrepancy between public and private services outcomes may be even more striking in Brazil. Therefore, our study showed an unfavorable impact on the clinical outcomes of patients treated in the public sector, even excluding the heterogeneity between the five regions that constitute Brazil.

Two large Brazilian studies, AMAZONA I and AMAZONA III, evaluated the scenario of breast cancer treatment in Brazil. The AMAZONA I assessed 4,912 patients retrospectively (2,198 diagnosed in 2001 and 2,714 diagnosed in 2006), and the AMAZONA III assessed 2,950 patients prospectively diagnosed between 2016 and 2018 (5, 15). Liedke et al., based on the AMAZONA I database, evaluated the differences in outcomes according to the type of healthcare coverage, public sector, or private sector (9). Considering the total study population, the OS of publicly insured patients was worse than that of privately insured patients. However, when stratifying into two clinical staging groups (0–II disease *versus* III–IV disease), OS difference was seen in patients with advanced staging (9). The oncological outcomes of the AMAZONA III study were not published due to a short follow-up, but this study demonstrated that in the public setting, more patients are diagnosed in the advanced stage (5). Similarly, in the present study, we showed a higher rate of advanced stages at diagnosis in publicly insured patients. Possibly, this might have impacted the OS outcomes of the assessed population. Additionally, the more advanced stage at diagnosis could explain the higher rate of conservative surgery performed in private compared to public practice.

In Brazil, there are scarce resources provided by the government and inefficient public–private collaboration (16). Additionally, admission to specialist care remains a major problem resulting in an immeasurable demand, delays in diagnosis, and long waiting times for treatment (17, 18). Nevertheless, the overall number of cases diagnosed in more advanced stages has decreased (19). A large population study carried out in Brazil with more than 193,000 breast cancer patients treated exclusively in the public health system (SUS) showed a reduction in the number of simple mastectomies and stable trends in radical mastectomy with lymphadenectomy (20).

Another difference seen between the two institutions, public and private, was the prevalence of ductal and lobular tumors. Li and colleagues published a study in the early 2000s that showed an increased prevalence of lobular tumors in a series of more than 190,000 breast cancer cases in the United States (21). Other studies showed that the prevalence of lobular carcinoma is approximately 15%, very similar to that found in the private scenario in our study (22, 23). Otherwise, the prevalence of lobular carcinomas in the public service is 6.8%, similar to the prevalence data for lobular carcinoma in the two largest series on breast cancer in Brazil, the AMAZONA I and AMAZONA III (5, 15).

Usually, patients with access to the private health system are treated according to the established standard of care supported by the main breast cancer guidelines. Differently, in the public health system, the timelines for new anticancer drug incorporation are extremely long. For instance, the first results of adjuvant treatment with trastuzumab were published between 2001 and 2005 (24–26). Before 2005, we had 61 patients from both sites with HER2-positive BC subtype, and none received this treatment. Only in 2013 was trastuzumab available for metastatic BC in the Brazilian public system; however, in some scenarios, BCH started to offer trastuzumab a couple of years before, in 2007 (27). Considering the estimated efficacy analysis of pivotal studies of anti-HER2 therapies, it was demonstrated that between 600 and 776 lives were lost per year due to lack of access to these drugs (27). In our study, we demonstrated that a higher proportion of patients in the private setting received anti-HER2 treatment. Therefore, our inclusion period between 2003 and 2011 leads to possible discrepancies in the oncological treatment performed, especially for HER2-positive BC patients.

Our study has some limitations, such as its retrospective nature leading to possible information or selection bias; the lack of adequate information between the time of diagnosis and the start of treatment, especially in the public service, which can influence oncological outcomes; the absence of sociodemographic analysis; and the comparison of two cohorts with very different sample sizes, with fewer participants from the private sector compared to the public sector. To minimize part of this last bias, we used the PSM, an adequate statistical tool to control confounding variables, especially in observational studies such as ours (28). By building an artificial control group using the quasi-experimental method of the PSM, we were able to reduce the disparity in the number of patients treated in the public system compared to the number of patients in the private system (29). Furthermore, it is not possible to infer that the difference in OS is related to the treatment offered at each center since the patients’ clinical characteristics were significantly different, particularly histological type and TNM staging. However, multivariate analyses with appropriate adjustments were conducted to reduce this possible sampling bias.

Comparing the results of two geographically remarkably close centers in a country with continental dimensions minimizes regional effects on the oncological outcomes, which is a strength of our study. In addition, the long-term follow-up allowed a mature analysis of OS and DFS.

Our study demonstrated that patients who arrive for treatment in the public service are at more advanced stages of breast cancer than those who arrive for treatment in the private service. This difficult access with delayed diagnosis leads to a lower number of conservative surgeries, a greater need for neoadjuvant therapy, less disease-free time, and higher mortality. However, the most important result of the study, when we adjusted the factors by disease stage, was that recurrence and mortality in the public service are higher than in the private service.

In conclusion, unfortunately, receiving breast cancer treatment in a private service in Brazil increases the chance of being alive compared to the same treatment in the public system. These outcomes demonstrated the worrisome treatment quality gap between two different healthcare systems located in the most populous state in Brazil. This is partly due to defective diagnostics landscapes and shortages, logistical and financial challenges such as high costs, complex regulatory pathways, and weak public–private partnerships. We urgently need to reduce sociocultural discrepancies in our country to improve cancer treatment for publicly insured patients.

## Data availability statement

The raw data supporting the conclusions of this article will be made available by the authors, without undue reservation.

## Ethics statement

The studies involving human participants were reviewed and approved by Barretos Cancer Hospital Ethical Review Board approved it in February 2017. Written informed consent for participation was not required for this study in accordance with the national legislation and the institutional requirements.

## Author contributions

DAPA and CPS were responsible for the conceptualization and supervision of the manuscript. The original manuscript was drafted by DAPA, ACV, CEP, RR, JPB, and CPS and subsequently reviewed and edited by all authors. CAFF, AONS, AWAB, ACMCP, GCMK, DDP, MCBB, MAS and GSFP contributed to analysis and interpretation of data writing, review and/or revision of the manuscript. All authors contributed to the article and approved the submitted version.

## Supplementary material

Supplementary Figure 1 Disease-free survival for stage I **(A)**, stage II **(B)** and stage III **(C)**.

Supplementary Figure 2 Overall survival for stage I **(A)**, stage II **(B)** and stage III **(C)**.
