# Supplementary figures and images for: Discrepancies in breast cancer’s oncological outcomes between public and private institutions in the southeast region of Brazil: a retrospective cohort study
Source: Front Oncol. 2023 Jun 27;13:1169982. doi: 10.3389/fonc.2023.1169982 (PMC10333566; doi:10.3389/fonc.2023.1169982)

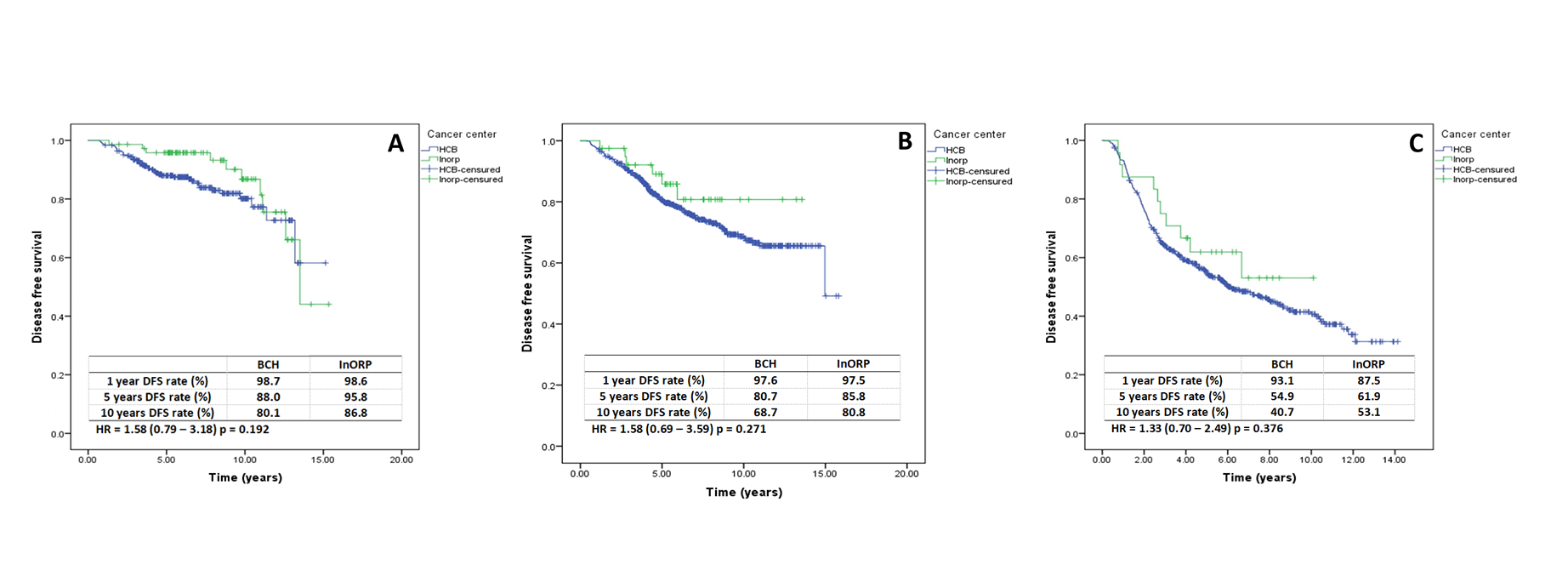

Supplement: Supplementary Figure 1 — Disease-free survival for stage I (A), stage II (B) and stage III (C). [file Image_1.tif]

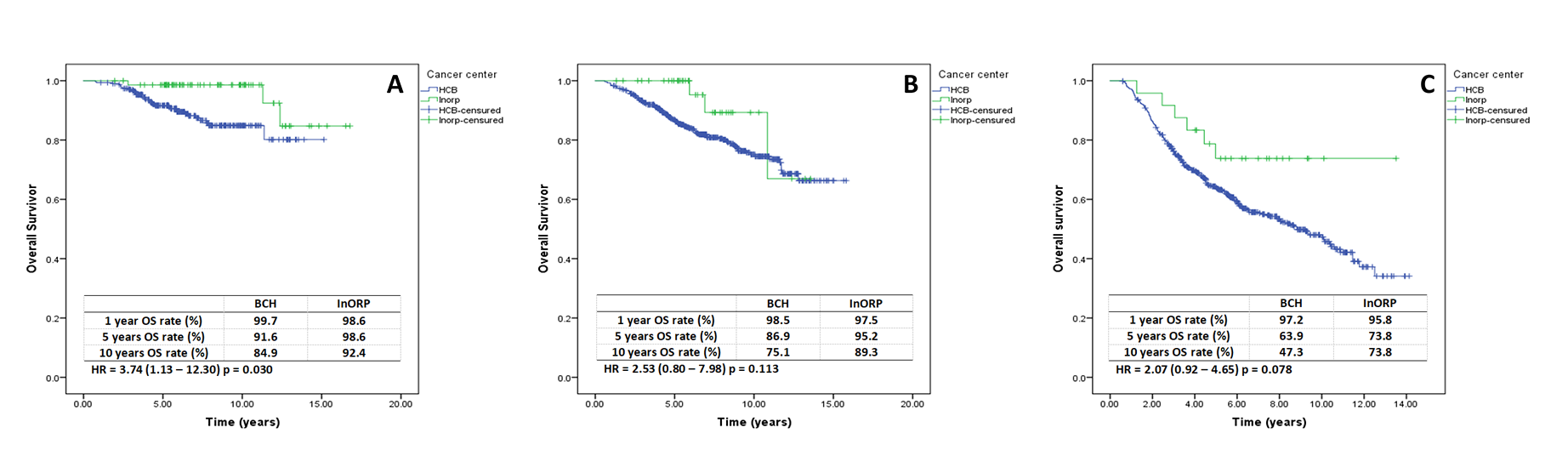

Supplement: Supplementary Figure 2 — Overall survival for stage I (A), stage II (B) and stage III (C). [file Image_2.tif]
